# Glycine at Position 622 in PB1 Contributes to the Virulence of H5N1 Avian Influenza Virus in Mice

**DOI:** 10.1128/JVI.02387-15

**Published:** 2016-01-28

**Authors:** Xiaoxiao Feng, Zeng Wang, Jianzhong Shi, Guohua Deng, Huihui Kong, Shiyu Tao, Changyao Li, Liling Liu, Yuntao Guan, Hualan Chen

**Affiliations:** State Key Laboratory of Veterinary Biotechnology, Harbin Veterinary Research Institute, Chinese Academy of Agricultural Sciences, Harbin, People's Republic of China

## Abstract

We isolated two H5N1 viruses, A/duck/Hunan/S4020/2008 (DK/08) and A/chicken/Guangxi/S2039/2009 (CK/09), from live-bird markets during routine surveillance and found that these two viruses are genetically similar but differ in their replication and virulence in mice. The CK/09 virus is lethal for mice with a 50% mouse lethal dose (MLD_50_) of 1.6 log_10_ 50% egg infectious doses (EID_50_), whereas the DK/08 virus is nonpathogenic for mice with an MLD_50_ value of 6.2 log_10_ EID_50_. We explored the genetic basis of the virulence difference of these two viruses by generating a series of reassortant viruses and mutants in the lethal virus CK/09 background and evaluating their virulence in mice. We found that the PB1 gene of the DK/08 virus dramatically attenuated the virulence of the CK/09 virus and that the amino acid at position 622 in PB1 made an important contribution. We further demonstrated that the mutation of glycine (G) to aspartic acid (D) at position 622 in PB1 partially impaired the binding of PB1 to viral RNA, thereby dramatically decreasing the polymerase activity and attenuating H5N1 virus virulence in mice. Our results identify a novel virulence-related marker of H5N1 influenza viruses and provide a new target for live attenuated vaccine development.

**IMPORTANCE** H5N1 avian influenza viruses have caused the deaths of nearly 60% of the humans that they have infected since 1997 and clearly represent a threat to public health. A thorough understanding of the genetic basis of virulence determinants will provide important insights for antiviral drug and live attenuated vaccine development. Several virulence-related markers in the PB2, PA, M1, and NS1 proteins of H5N1 viruses have been identified. In this study, we isolated two H5N1 avian influenza viruses that are genetically similar but differ in their virulence in mice, and we identified a new virulence-related marker in the PB1 gene. We found that the mutation of glycine (G) to aspartic acid (D) at position 622 in PB1 partially impairs the binding of PB1 to viral RNA, thereby attenuating H5N1 virus virulence in mice. This newly identified virulence-related marker could be applied to the development of live attenuated vaccines against H5N1 influenza.

## INTRODUCTION

H5N1 avian influenza outbreaks in poultry have become widespread since late 2003, and H5N1 viruses have caused numerous disease outbreaks in domestic poultry and wild birds in many countries throughout Asia, Europe, and Africa (http://www.oie.int). H5N1 virus infection of humans has been reported in 16 countries, with 429 deaths among 784 cases as of 3 March 2015 (http://www.who.int). Several studies have shown that the H5N1 viruses could become transmissible in mammals if they acquired more mutations or reassorted with human influenza viruses (1–5). Thus, the H5N1 viruses circulating in nature pose huge threats to both animals and public health.

The influenza A virus genome comprises eight gene segments, including basic polymerase 2 (Pol II; PB2 gene), basic polymerase 1 (PB1 gene), acidic polymerase (PA gene), hemagglutinin (HA gene), nucleoprotein (NP gene), neuraminidase (NA gene), matrix (M gene), and nonstructural protein (NS gene). These gene segments encode at least 12 proteins, including PB2, PB1, PB1-F2, PA, PA-X, HA, NA, NP, M1, M2, NS1, and NS2. Many studies have investigated the molecular basis of the lethality of H5N1 avian influenza viruses in mammalian hosts, and a series of virulence-related amino acids in different proteins have been identified (6–17). For example, in the PB2 protein, the amino acid substitution at position 627 from glutamic acid to lysine (E627K) and the amino acid substitution at position 701 from aspartic acid to asparagine (D701N) play crucial roles in the ability of H5N1 viruses to replicate and be lethal in mammals (1, 6). Several amino acids in the PA proteins, including those at positions 97, 185, 224, and 383, have been reported to affect the virulence of H5N1 viruses in mice (12, 15, 17). The motif of multiple basic amino acids in the cleavage site of HA is the prerequisite for the virulence of H5N1 virus in both avian and mammalian hosts (6, 18). The amino acids asparagine at position 30 and alanine at position 215 in the M1 protein are necessary for H5N1 virus lethality in mice (13). Moreover, the amino acid serine at position 42 of NS1 is critical for the H5N1 influenza virus to antagonize host cell interferon induction and for the pathogenicity of H5N1 influenza viruses in mammalian hosts (11).

During our routine surveillance studies, we isolated two H5N1 viruses, A/duck/Hunan/S4020/2008 (DK/08) and A/chicken/Guangxi/S2039/2009 (CK/09), from birds in live-bird markets and found that these two viruses are genetically similar but differ in their replication and virulence in mice. Yet all of the known virulence-related markers are the same in these two viruses, suggesting that other amino acids or motifs may contribute to the difference in lethality between these two viruses in mice. In this study, we used reverse genetics to generate a series of reassortants and mutants of these two viruses in the CK/09 background and tested their virulence in mice. We identified a novel virulence-related markers in the PB1 protein and explored the underlying mechanism for the difference in lethality between these two viruses in mice.

## MATERIALS AND METHODS

### Ethics statement and facility.

The present study was carried out in strict accordance with the recommendations in the Guide for the Care and Use of Laboratory Animals of the Ministry of Science and Technology of the People's Republic of China. Studies with highly pathogenic H5N1 avian influenza viruses were conducted in a biosecurity level 3 laboratory approved for such use by the Chinese Ministry of Agriculture. The protocol was approved by the Committee on the Ethics of Animal Experiments of the Harbin Veterinary Research Institute (HVRI) of the Chinese Academy of Agricultural Sciences (CAAS).

### Cells and viruses.

Human embryonic kidney cells (293T) and MDCK cells incubated at 37°C in 5% CO_2_ were grown in Dulbecco's modified Eagle's medium supplemented with 10% and 5% fetal bovine serum, respectively, plus antibiotics. The H5N1 viruses, A/duck/Hunan/S4020/2008 (DK/08) and A/chicken/Guangxi/S2039/2009 (CK/09), were isolated from live-bird markets during routine surveillance. Virus stocks were propagated in 10-day-old specific-pathogen-free (SPF) embryonated chicken eggs and stored at −70°C until they were used for RNA extraction and animal studies.

### Sequence analysis.

Viral RNA (vRNA) was extracted from allantoic fluid and was subjected to reverse transcription (RT). A set of fragment-specific primers (primer sequences available on request) were used for the PCR amplification and sequence analysis.

### Construction of plasmids for virus rescue.

Construction of plasmids in viral RNA vRNA-mRNA bidirectional expression plasmid pBD for virus rescue was performed as described previously (8) with the primers shown in Table 1. The constructs were designated pBD/DK/08-PB2, pBD/DK/08-PB1, pBD/DK/08-PA, pBD/DK/08-HA, pBD/DK/08-NP, pBD/DK/08-NA, pBD/DK/08-M, pBD/DK/08-NS, pBD/CK/09-PB2, pBD/CK/09-PB1, pBD/CK/09-PA, pBD/CK/09-HA, pBD/CK/09-NP, pBD/CK/09-NA, pBD/CK/09-M, and pBD/CK/09-NS, respectively. Mutations were introduced into the PB1 gene by site-directed mutagenesis (Invitrogen) with the primers shown in Table 1. All of the constructs were completely sequenced to ensure the absence of unwanted mutations.

**TABLE 1:** Primers used for pBD cDNA construction and for introducing mutations into the PB1 gene of the mutant viruses

Purpose	Primer(s) (5′–3′)^*a*^	
	Forward	Reverse
PB2 amplification	CCAGCAAAAGCAGGTCAAATATATTCA	TTAGTAGAAACAAGGTCGTTTTTAAAT (DK/08), TTAGTAGAAACAAGGTCGTTTTTAAAC (CK/09)
PB1 amplification	CCAGCAAAAGCAGGCAAACCATTTGAATG	TTAGTAGAAACAAGGCATTTTTTCACG
PA amplification	CCAGCAAAAGCAGGTACTGATCCAAA	TTAGTAGAAACAAGGTACTTTTTTGGA
HA amplification	CCAGCAAAAGCAGGGGTTCACTCTGTC (DK/08), CCAGCAAAAGCAGGGGTCCAATCTGTC (CK/09)	TTAGTAGAAACAAGGGTGTTTTTAACTAC
NP amplification	CCAGCAAAAGCAGGGTAGATAATCAC	TTAGTAGAAACAAGGGTATTTTTCT
NA amplification	CCAGCAAAAGCAGGAGTTCA	TTAGTAGAAACAAGGAGT
M amplification	CCAGCAAAAGCAGGTAGATGT	TTAGTAGAAACAAGGTAGTTT
NS amplification	CCAGCAAAAGCAGGGTGACAAAAACAT	TTAGTAGAAACAAGGGTGTTTTTTA
CK/09 PB1D619N mutation	GAAATGGGAATTGATGGATGAA**A**ACTACCAGGGCAG	CTGCCCTGGTAGT**T**TTCATCCATCAATTCCCATTTC
CK/09 PB1G622D mutation	GGATGAAGACTACCAGG**A**CAGACTGTGCAATCCTC	GAGGATTGCACAGTCTG**T**CCTGGTAGTCTTCATCC
CK/09 PB1R635K mutation	CTGAATCCATTCGTCAGCCATA**A**GGAAATTGAATCTGTC	GACAGATTCAATTTCC**T**TATGGCTGACGAATGGATTCAG
CK/09 PB1D619N/G622D mutation	GGATGAA**A**ACTACCAGG**A**CAGACTGTGCAATCCTC	GAGGATTGCACAGTCTG**T**CCTGGTAGT**T**TTCATCC

a The nucleotides that have been changed are underlined and in boldface type.

### Generation of reassortant viruses.

Reassortant or mutant viruses were generated by use of reverse genetics as described previously (19–21). The rescued viruses were detected by using a hemagglutination assay, and RNA was extracted and analyzed by reverse transcription-PCR (RT-PCR). Each viral segment was sequenced to confirm the identity of the reassortant viruses.

### Animal experiments.

For the replication study, groups of three 6-week-old female BALB/c mice (Beijing Experimental Animal Center) were lightly anesthetized with CO_2_ and inoculated intranasally with 10^6.0^ 50% egg infectious doses (EID_50_) of H5N1 influenza virus in a volume of 50 μl and were euthanized on day 3 postinoculation (p.i.). Lungs, brains, kidneys, and spleens of mice were collected and titrated for virus infectivity in eggs as described previously (22). The 50% mouse lethal dose (MLD_50_) was determined by inoculating groups of five 6-week-old female BALB/c mice with 10-fold serial dilutions containing 10^1^ to 10^6^ EID_50_ of the virus in a 50-μl volume.

### Polymerase activity.

A dual-luciferase reporter assay system (Promega) was used to compare the activities of viral ribonucleoprotein (RNP) complexes. To construct the reporter plasmid, pPolI-Luc, the open reading frame of the luciferase gene, flanked by the 5′ and 3′ noncoding regions of the NP gene of the DK/08 virus, was inserted into a pPol I plasmid containing the sequence of the human polymerase I promoter. Briefly, 0.5 μg of luciferase reporter plasmid pPolI-Luc and the Renilla luciferase expressing plasmid pTK-RL was transfected into 2 × 10^5^ 293T cells together with 0.5 μg of each of the four protein expression plasmids pcDNA3.1-PB2, pcDNA3.1-PB1 (or PB1 mutants), pcDNA3.1-PA, and pcDNA3.1-NP of CK/09 or DK/08. The Renilla luciferase expressed by pTK-RL was used as an internal control to normalize transfection efficiency. Cell extracts were harvested 30 h posttransfection, and luciferase activity was assayed by using the luciferase assay system (Promega). The assay was standardized to the activity of CK/09 (100%). There were two replicates for each complex combination each time, and all experiments were performed three times.

### Viral replication in MDCK cells.

Virus was inoculated into MDCK monolayers at a multiplicity of infection (MOI) of 0.001. The cells were supplemented with Opti-MEM and incubated at 37°C. Virus-containing culture supernatant was collected at various time points (hours postinfection [hpi]) and titrated in MDCK cells. The growth data shown are the average results of three independent experiments.

### Generation of the model vRNA.

A 290-nucleotide model vRNA was transcribed *in vitro* by using a cDNA containing the T7 promoter that was amplified by overlapping PCR with two sets of primers (primer set NS-F1 [5′AGCAAAAGCAGGGTGACAAAAACAT] and NS-R171 [ctaggctgaccgTGTTGCCTCTTCCTCTTAGTGACTT] and primer set NS-F769 [cggtcagcctagCAGATAACGTTTATGCAAGCCTTAC] and NS-R875 [taatacgactcactataggAGTAGAAACAAGGGTGTT]) (the lowercase nucleotides represent the sequence of the T7 promoter) from the CK/09-NS plasmid pBD/CK/09-NS template.

### Protein binding vRNA assay.

293T cells were transfected with a plasmid expressing Flag-tagged, truncated CK/09-PB1Δ1-493 or with CK/09-PB1Δ1-493/G622D or with plasmid pCAGGS-3Flags as a control. The cells were lysed in radioimmunoprecipitation assay (RIPA) lysis buffer (Thermo) (25 mM Tris·HCl [pH 7.6], 150 mM NaCl, 1% NP-40, 1% sodium deoxycholate, 0.1% SDS) supplemented with 1× proteinase inhibitors. Cell lysate containing 50 μg of total protein was added to protein G (Life Technology) that had been cross-linked with a mouse anti-Flag monoclonal antibody and were incubated for 4 h on a roller at 4°C. The magnetic beads and their captured proteins were precipitated onto a magnetic stand, and the supernatant was discarded. After three washes with diethyl pyrocarbonate (DEPC)-treated 0.1 M NaCl, the captured proteins were mixed with 10 μg of vRNA in a total volume of 200 μl and incubated on a roller for 4 h at 4°C. The supernatant containing the unbound vRNA and the magnetic beads bearing the protein-vRNA mixture were separated by using the magnetic stand. Both the unbound and protein-bound vRNAs were quantified by means of real-time RT-PCR (primer sequences available on request).

### vRNA binding protein assay.

293T cells were transfected with a plasmid expressing Flag-tagged, truncated CK/09-PB1Δ1-493 or with CK/09-PB1Δ1-493/G622D or with plasmid pCAGGS-Flag as a control. The cells were lysed in RIPA lysis buffer (Thermo) (25 mM Tris·HCl [pH 7.6], 150 mM NaCl, 1% NP-40, 1% sodium deoxycholate, 0.1% SDS) supplemented with 1× proteinase inhibitors. Ten micrograms of model vRNA was labeled with 10 μg of photobiotin under strong light (450 W, 220 V) for 30 min. The biotinylated vRNA was then incubated with cell lysate containing 150 μg of total protein in RIPA buffer (25 mM Tris [pH 7.4], 150 mM KCl, 5 mM EDTA, 0.5 mM dithiothreitol [DTT], 0.5% Nonidet P-40) supplemented with 100 U/ml RNase inhibitor RNaseOUT (Invitrogen) and 1× proteinase inhibitors (Roche), in a total volume of 400 μl, on a roller for 4 h at 4°C. The biotinylated RNA/protein complexes were precipitated with Dynabeads M-280 streptavidin (Invitrogen) on a magnetic stand. The supernatants were harvested for subsequent Western blotting. The precipitated complexes were washed 5 times with ice-cold washing buffer (5 mM Tris-HCl [pH 7.5], 0.5 mM EDTA, 1 M NaCl) and resolved by means of 15% SDS-PAGE followed by Western blotting.

### Western blotting.

The cell lysate proteins captured by protein G, unbound proteins, and vRNA-bound proteins from the vRNA binding protein assay were resolved by use of 15% SDS-PAGE and transferred to a nitrocellulose membrane (Pall). The membrane was blocked in 5% skim milk–Tris-buffered saline–Tween 20 (TBST) buffer for 45 min at room temperature and then probed with an anti-Flag mouse monoclonal antibody (Sigma) for 2 to 4 h at room temperature. After hybridization with a goat anti-mouse secondary antibody (KPL), the membranes were visualized with enhanced chemiluminescence reagents (Pierce).

### Nucleotide sequence accession numbers.

The sequence data for the two viruses used in these studies are available in GenBank (accession numbers KT762428 to KT762443).

## RESULTS

### Virus rescue and characterization.

We cloned the cDNAs of each full-length RNA segment of the DK/08 and CK/09 viruses into a vRNA-mRNA bidirectional expression plasmid (pBD) as described previously (8) and by using the set of gene segment-specific primers shown in Table 1. All of the constructs were completely sequenced to ensure the absence of unwanted mutations. By using these plasmids, we rescued the DK/08 and CK/09 viruses, designated R-DK/08 and R-CK/09, respectively, grew them in 10-day-old SPF embryonated chicken eggs, and tested their replication and lethality in mice. The rescued R-DK/08 virus, like the wild-type DK/08, replicated only in the lungs and killed only one mouse at the inoculated dose (10^6^ EID_50_), which was administered intranasally, yielding a 50% MLD_50_ of 6.2 log_10_ EID_50_ (Table 2). However, R-CK/09 replicated systemically and was as highly pathogenic as the original CK/09 virus, with an MLD_50_ of 1.5 log_10_ EID_50_ (Table 2). These results indicate that the rescued viruses maintained the biological properties of the wild-type viruses.

**TABLE 2:** Replication and lethality of H5N1 viruses in mice^*a*^

Virus	Virus replication on day 3 p.i. (log_10_ EID_50_ ± SD)^*b*^				MLD_50_ (log_10_ EID_50_)^*c*^	Attenuation (fold)^*f*^
	Lung	Brain	Spleen	Kidney		
DK/08	6.0 ± 0.5	−	−	−	6.2	NA
CK/09	6.7 ± 0.5	3.8 ± 0.6	4.2 ± 0.4	4.0 ± 0.7	1.6	NA
R-DK/08	6.0 ± 0.7	−	−	−	6.2	NA
R-CK/09	7.3 ± 0.1	4.5 ± 0.0	4.5 ± 0.0	4.4 ± 0.1	1.5	NA
CK/09-DK/08PB2	6.5 ± 0.7	4.5 ± 0.0	4.1 ± 0.7	4.4 ± 0.1	1.4	0
CK/09-DK/08PB1	5.3 ± 0.4^*d*^	−	1.5 ± 0.0^*d*^	−	4.5	1,000
CK/09-DK/08PA	6.5 ± 0.3	4.2 ± 0.4	4.2 ± 0.6	3.8 ± 0.7	1.2	0
CK/09-DK/08HA	6.8 ± 0.4	3.9 ± 0.5	4.5 ± 0.0	4.2 ± 0.6	1.3	0
CK/09-DK/08NP	6.6 ± 0.8	4.4 ± 0.1	4.4 ± 0.1	4.3 ± 0.1	1.2	0
CK/09-DK/08NA	7.1 ± 0.5	4.5 ± 0.0	4.4 ± 0.1	4.5 ± 0.0	1.2	0
CK/09-DK/08 M	6.8 ± 0.6	3.4 ± 0.8	4.4 ± 0.1	4.1 ± 0.5	1.4	0
CK/09-DK/08NS	6.7 ± 0.3	4.1 ± 0.5	4.5 ± 0.0	4.0 ± 0.7	1.2	0
Chimera 1	6.3 ± 0.4	3.5 ± 0.9	4.4 ± 0.1	4.2 ± 0.6	1.8	2
Chimera 2	4.9 ± 0.3^*d*^	−	1.4 ± 1.0^*d*^	1.3 ± 0.9^*d*^	4.5	1,000
CK/09-PB1D619N	6.6 ± 0.1	2.9 ± 1.4	4.0 ± 0.4	3.8 ± 0.4	1.4	0
CK/09-PB1G622D	5.8 ± 0.5^*d*^	1.2 ± 0.8^*d*^	2.9 ± 2.1^*d*^	2.0 ± 1.3^*d*^	4.2	501
CK/09-PB1R635K	6.5 ± 0.2	3.3 ± 0.9	3.6 ± 0.6	3.6 ± 0.9	2.5	10
CK/09-PB1D619N+G622D	5.3 ± 0.4	−	2.3 ± 0.3^*d*^	−	4.5	1,000
CK/09-PB1G622D+R635K	3.8 ± 0.6^*e*^	−	3.3 ± 0.5	1.3 ± 1.0^*e*^	4.2	501

a Six-week-old female BALB/c mice were used for these studies.

b Groups of three mice were inoculated intranasally with 10^6^ EID_50_ of the test virus in a 50-μl volume and were killed on day 3 postinoculation (p.i.); organs were then collected for virus titration in eggs. −, no virus was detected in undiluted samples. Virus titers of mice were compared by using the Student-Newman-Keuls test. SD, standard deviation.

c The 50% mouse lethal dose (MLD_50_) was determined by intranasally inoculating groups of five mice with 10-fold serial dilutions containing 10^1^ to 10^6^ EID_50_ of virus in a 50-μl volume.

d *P* < 0.05 compared with the titers in the corresponding organs of the R-CK/09-inoculated mice.

e *P* < 0.01 compared with the titers in the corresponding organs of the R-CK/09-inoculated mice.

f Virulence decrease compared with the R-CK/09 virus. NA, not applicable.

### The PB1 gene plays a major role in the difference in pathogenicity in mice between the DK/08 and CK/09 viruses.

**FIG 1:**
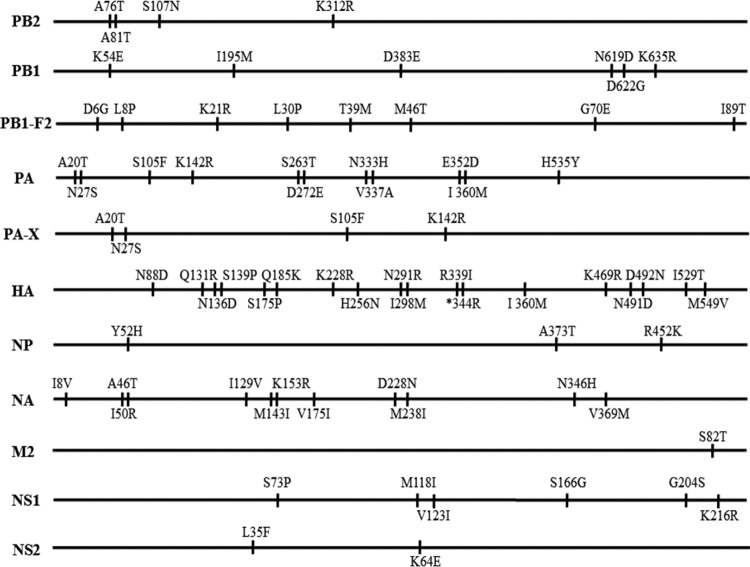
Amino acid differences between the DK/08 and CK/09 viruses. The amino acid differences between the two viruses are shown as single letters at the indicated positions. Each amino acid of DK/08 is shown before the number of the position, and each amino acid of CK/09 is shown after the number of the position. *, amino acid deletion at position 344 in the HA of DK/08 virus.

The two viruses differ by 74 amino acids in their 11 proteins (Fig. 1). We used the “single-gene recombinant” strategy to identify genes that contributed to the virulence of the viruses in mice, as described elsewhere (6, 8–16). However, to avoid any “gain-of-function” concerns, we used only the CK/09 lethal virus as the backbone to generate the reassortants, each containing one gene derived from DK/08, and tested their replication and pathogenicity in mice. Seven viruses that carried the PB2, PA, HA, NP, NA, M, or NS gene of DK/08 virus were as virulent as the CK/09 virus and replicated in all four mouse organs tested (Table 2). However, introduction of the PB1 gene of the DK/08 virus dramatically attenuated the pathogenicity of CK/09 by 1,000-fold (MLD_50_ of 1.5 versus 4.5 log_10_ EID_50_), and the reassortant virus replicated only in the lungs and spleen of infected mice (Table 2).

### The amino acid glycine at position 622 of PB1 is critical for the virulence of the CK/09 virus in mice.

**FIG 2:**
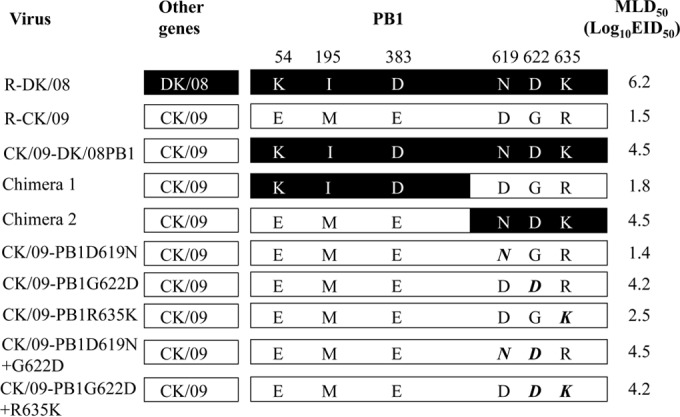
PB1 mutants of R-CK/09 and their virulence in mice. The bars show the origin of the genes as follows: black, DK/08; white, CK/09. The amino acid differences between the PB1 protein of DK/08 and that of CK/09 are shown as single letters with their positions numbered at the top, and the mutated amino acids are shown in boldface italic type. The MLD_50_ was determined by intranasally inoculating groups of five mice with 10-fold serial dilutions containing 10^1^ to 10^6^ EID_50_ of the virus in a 50-μl volume. The 506 amino acids in the N-terminal of PB1 in the chimera 1 virus and the 251 amino acids in the C-terminal of PB1 of the chimera 2 virus were derived from the DK/08 virus.

The PB1 proteins of the DK/08 and CK/09 viruses differ by six amino acids (Fig. 2). To pinpoint the amino acid(s) in PB1 that contributes to the virulence of the CK/09 virus, we generated two viruses that expressed chimeric PB1 proteins (Fig. 2) and tested them in mice. When the N-terminal portion of the CK/09 PB1 gene was replaced with the corresponding DK/08 PB1 gene segment (chimera 1), within the context of the remaining genes coming from CK/09, the chimeric virus was as lethal as the wild-type CK/09 virus and replicated systemically in mice (Table 2 and Fig. 2). However, chimera 2 (the CK/09 virus with the C-terminal portion of the DK/08 PB1 gene) was attenuated 1,000-fold, and the virus replicated in multiple organs, but the titers were lower than those of the wild-type CK/09 virus (Table 2). These results indicate that the C terminus of the PB1 gene is important for the virulence of the CK/09 virus in mice.

The two viruses differ by only three amino acids in this region of PB1 at positions 619, 622, and 635 (Fig. 2). To pinpoint which amino acid contributes to the pathogenicity in mice, we generated and tested three mutants, CK/09-PB1D619N, CK/09-PB1G622D, and CK/09-PB1R635K (Fig. 2). All three mutants were detected in all four organs tested in mice, but the titers of CK/09-PB1G622D virus were significantly decreased and the virulence of the mutant was attenuated over 500-fold compared with that of the R-CK/09 virus (Table 2). Addition of the D619N substitution slightly attenuated the CK/09-PB1G622D virus results (MLD_50_, 4.5 log_10_ EID_50_ versus 4.2 log_10_ EID_50_), and the virus was not detected in the brains or kidneys of mice inoculated with this double mutant. The combination of this mutation with the mutation at position 635 of PB1 did not further attenuate the CK/09-PB1G622D virus results in mice (Table 2 and Fig. 2). These data indicate that the glycine amino acid at position 622 of PB1 is essential for the virulence of the CK/09 virus in mice, although it is not entirely responsible for the virulence difference between these two viruses.

### The amino acid at position 622 in the PB1 protein affects viral polymerase activity.

**FIG 3:**
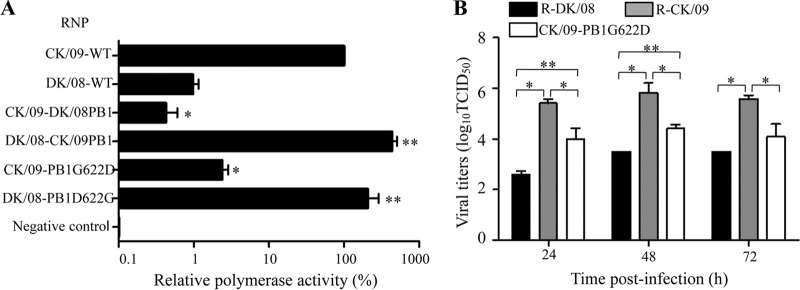
Mutation at position 622 in the PB1 protein affects the polymerase activity and replication of H5N1 viruses *in vitro*. (A) Polymerase activities of the DK/08 and CK/09 RNP complexes with different PB1 mutations in a minigenome assay. The values shown are means ± standard deviations of results for three independent experiments and are standardized to the activity of CK/09 (100%). *, *P* < 0.01 (compared with the results seen with CK/09 RNP-transfected cells); **, *P* < 0.01 (compared with the results seen with DK/08 RNP-transfected cells). WT, wild type. (B) Multicycle replication of H5N1 avian influenza viruses in MDCK cells. MDCK monolayers were inoculated at an MOI of 0.001 with virus, and the culture supernatants were collected at the indicated time points and then titrated in MDCK cells. *, *P* < 0.01 (compared with titers in CK/09 virus-infected cells); **, *P* < 0.01 (compared with titers in DK/08 virus-infected cells). The Student-Newman-Keuls test was used for the statistical analysis.

The PB1 protein is a component of the RNP complex, which plays an important role in influenza virus replication and virulence (5, 23–25). We compared the polymerase activities of the DK/08 and CK/09 viruses in a minigenome assay in 293T cells as described previously (14). The polymerase activity of the RNP complex of the CK/09 virus was more than 100-fold greater than that of the DK/08 virus, and exchange of the PB1 gene in the RNP complex dramatically altered their activities (Fig. 3A). Moreover, introduction of the G622D mutation in PB1 significantly decreased the activity of the RNP complex of the CK/09 virus, whereas the D622G mutation in PB1 significantly increased the activity of the RNP complex of the DK/08 virus (Fig. 3A).

### The amino acid at position 622 in the PB1 protein affects viral replication in MDCK cells.

We compared the multicycle growth levels of R-DK/08, R-CK/09, and CK/09-PB1G622D in MDCK cells. The R-CK/09 virus grew more rapidly than did the R-DK/08 virus, and the titers of R-CK/09 were significantly higher than those of R-DK/08 (Fig. 3B). The titers of CK/09-PB1G622D were significantly lower than those of R-CK/09 but were higher than those of R-DK/08 (Fig. 3B), which was in agreement with the replication and virulence in mice of these viruses.

### The G622D mutation in PB1 partially impairs the ability of PB1 to bind vRNA.

**FIG 4:**
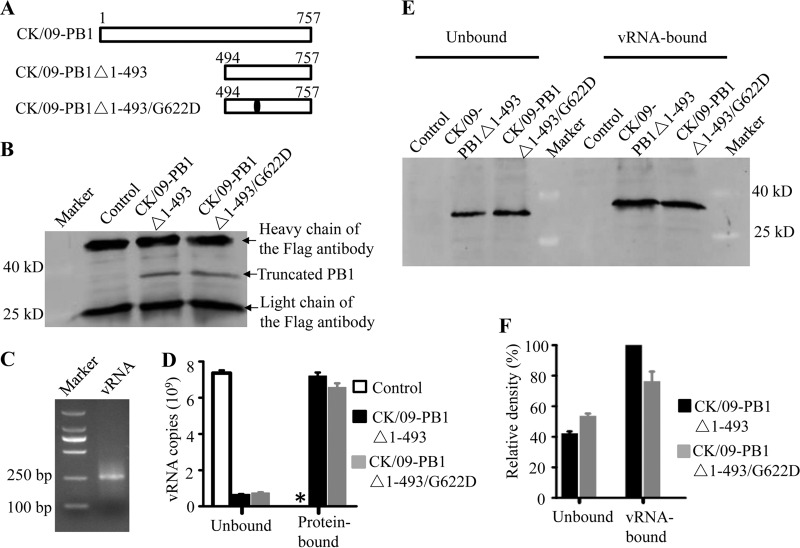
Truncated PB1 and vRNA binding assays. (A) 293T cells were transfected with plasmid pCAGGS-3Flags-PB1Δ1-493, which expresses the Flag-tagged, truncated CK/09-PB1Δ1-493 protein, or with plasmid pCAGGS-3Flags-PB1Δ1-493/G622D, which expresses the CK/09-PB1Δ1-493/G622D protein, or with plasmid pCAGGS-3Flags as a control. Cell lysates containing 100 μg of total protein were captured by analysis using protein G (Life Technology). (B) The capture of the PB1 proteins was confirmed by use of Western blotting. (C) The protein G-captured proteins were also used to bind model vRNA, which was transcribed *in vitro*. (D) The vRNA bound by different proteins was quantified by use of real-time RT-PCR. (E and F) vRNA binding to different truncated PB1 proteins was also assayed as described in Materials and Methods; 20% of the unbound protein and all of the vRNA-bound protein were used for SDS-PAGE followed by Western blotting (E), and the density of each band was scaled by using an Infrared Imaging System (Odyssey) and compared with that of vRNA-bound CK/09-PB1Δ1-493 protein (F). *, the vRNA was undetectable. The data shown in panels D and F are the means of the results of three experiments; the bars show the standard deviations.

PB1 has two regions (the N-terminal 83 amino acids and the C-proximal sequences located downstream of position 493) that bind to vRNA (26–30). The amino acid at position 622 is located in one of the vRNA binding domains of PB1. We therefore tested whether the G622D mutation affects the binding of PB1 to vRNA. 293T cells were transfected with plasmid pCAGGS-3Flags-PB1Δ1-493, which expresses the Flag-tagged, truncated CK/09-PB1Δ1-493 protein, or with plasmid pCAGGS-3Flags-PB1Δ1-493/G622D, which expresses the CK/09-PB1Δ1-493/G622D protein (Fig. 4A), or with plasmid pCAGGS-3Flags as a control. Cell lysates containing 50 μg of total protein were captured by protein G (Life Technology) that had been cross-linked with a mouse anti-Flag monoclonal antibody. The captured PB1 proteins (Fig. 4B) were then used to bind 10 μg of model vRNA that was transcribed *in vitro* (Fig. 4C). After a 4-h incubation at 4°C, the unbound and protein-bound vRNA was quantified by use of real-time RT-PCR. As shown in Fig. 4D, the amount of vRNA bound by CK/09-PB1Δ1-493/G622D was about 90% of that bound by CK/09-PB1Δ1-493. In the plasmid pCAGGS-3Flags-transfected cell lysate, the truncated PB1 protein was not captured (Fig. 4B) and binding of vRNA was not detected (Fig. 4D).

We also performed a vRNA binding protein assay. Ten micrograms of biotinylated model vRNA was incubated with cell lysate containing 150 μg of total protein that had previously been transfected with plasmid pCAGGS-3Flags-PB1Δ1-493 or plasmid pCAGGS-3Flags-PB1Δ1-493/G622D or with plasmid pCAGGS-3Flags as a control. Both the unbound and vRNA-bound proteins were resolved by means of SDS-PAGE followed by Western blotting. We found that the vRNA did not bind any protein in the pCAGGS-3Flags plasmid-transfected cell lysate (Fig. 4E) and that the same amount of vRNA bound to more CK/09-PB1Δ1-493 protein than CK/09-PB1Δ1-493/G622D protein (Fig. 4E and F). Therefore, the results of these two assays, the protein binding vRNA assay and the vRNA binding protein assay, were in accord and demonstrated that the G622D mutation in PB1 partially impairs the ability of PB1 to bind vRNA.

## DISCUSSION

Here, we characterized two H5N1 avian influenza viruses, DK/08 and CK/09, which have similar genomes but show markedly different pathogenicities in mice. Using this pair of viruses and single-gene-reassortant viruses created from them, we demonstrated that the PB1 protein contributes to the different pathogenicities of these two viruses in mice and that the amino acid residue at position 622 in PB1 plays an important role in the virulence of H5N1 virus in mice. We further demonstrated that the G622D mutation partially impairs the binding of PB1 to vRNA and thereby attenuates the polymerase activity and virulence of the H5N1 virus.

PB1 has several functional domains; its N and C termini interact with PA and PB2, respectively (26, 29–37), and PB1 contains two regions that bind to vRNA (26–30). The amino acid at position 622 is located in one of these vRNA binding domains. The G622D mutation in PB1 resulted in a 10% reduction in the ability of PB1 to bind vRNA *in vitro* but in a 40-fold decrease in polymerase activity and a 500-fold attenuation of the virulence of the virus in mice. The 10% reduction of PB1/622D binding to vRNA observed *in vitro* reflects a single round of PB1-vRNA binding; however, the reduction in binding is greater *in vivo*, as reflected in the polymerase activity analysis and animal studies, because multiple rounds of PB1-vRNA binding would have occurred. The G622D mutation may also alter the interaction of PB1 with PB2 or PA, although this seems unlikely because the PB1 N-terminal PA-interacting helix (residues 1 to 15) and the C-terminal three-helix bundle (residues 685 to 757) that interacts with residues 1 to 35 of PB2 (35, 38) are located far from the amino acid at position 622. Further studies are warranted to determine whether the G622D mutation in PB1 alters its interaction with host proteins.

Many studies have been undertaken to identify the virulence determinants of influenza viruses in different hosts. These studies have identified a number of virulence-related markers by using different virus strains (1, 11, 15, 17, 39, 40). However, the underlying mechanisms of only a few of these markers have been revealed. The D701N mutation in PB2 is reported to have a key role in increasing the replication and virulence of different avian influenza viruses in mammals (8). This mutation disrupts the salt bridge with Arg753 of the nuclear localization site of PB2 and, thereby, human importin α5 binding and, in turn, the efficiency of trimeric polymerase assembly (41). Zhu et al. found that deletion of the amino acids at positions 191 to 195 in NS1 attenuates H5N1 influenza virus in chickens by affecting the stability of the NS1 protein and impairing the interaction of NS1 protein with chicken cleavage and polyadenylation specificity factor (40). Jiao et al. further demonstrated that the amino acid serine at position 42 of NS1 is essential for H5N1 influenza virus to antagonize host cell interferon induction by preventing the double-stranded RNA-mediated activation of the NF-κB pathway and the interferon regulatory transcription factor 3 (IRF-3) pathway (11). The present study showed that the G622D mutation in PB1 attenuates the H5N1 influenza virus in mice by partially impairing the binding of PB1 to vRNA. These findings indicate that different genetic changes in influenza virus alter its virulence through different mechanisms.

Although, according to the available sequences in the public database, the glycine at position 622 in PB1 is highly conserved among different subtypes of influenza viruses, a few strains have been detected in nature that have 622D in their PB1 (Table 3). We sequenced the viruses that were recovered from the mouse organs and found that wild-type DK/08 and the mutants generated in this study stably maintained 622D in their PB1 after they replicated in mice. Thus, the G622D mutation in PB1 could potentially be used in the development of live attenuated vaccines against influenza viruses.

**TABLE 3:** Naturally isolated influenza viruses bearing 622D in their PB1 sequence

Virus	Accession no. of PB1 sequence in GenBank
A/mallard/Sweden/68561/2007 (H4N6)	CY165168
A/swine/England/WVL16/1998 (H1N1)	CY037950
A/swine/England/26029/1998 (H1N1)	CY116072
A/swine/England/WVL15/1997 (H1N1)	CY037942
A/swine/England/636804/1996 (H1N1)	CY116250
A/swine/England/167655/1997 (H1N1)	CY116002
A/duck/Hunan/S4020/2008 (H5N1)	KT762429

## References

[1] Gao Y, Zhang Y, Shinya K, Deng G, Jiang Y, Li Z, Guan Y, Tian G, Li Y, Shi J, Liu L, Zeng X, Bu Z, Xia X, Kawaoka Y, Chen H 2009 Identification of amino acids in HA and PB2 critical for the transmission of H5N1 avian influenza viruses in a mammalian host. PLoS Pathog 5:e1000709. doi:10.1371/journal.ppat.1000709. 20041223 PMC2791199

[2] Chen LM, Blixt O, Stevens J, Lipatov AS, Davis CT, Collins BE, Cox NJ, Paulson JC, Donis RO 2012 In vitro evolution of H5N1 avian influenza virus toward human-type receptor specificity. Virology 422:105–113. doi:10.1016/j.virol.2011.10.006. 22056389 PMC5480292

[3] Herfst S, Schrauwen EJ, Linster M, Chutinimitkul S, de Wit E, Munster VJ, Sorrell EM, Bestebroer TM, Burke DF, Smith DJ, Rimmelzwaan GF, Osterhaus AD, Fouchier RA 2012 Airborne transmission of influenza A/H5N1 virus between ferrets. Science 336:1534–1541. doi:10.1126/science.1213362. 22723413 PMC4810786

[4] Imai M, Watanabe T, Hatta M, Das SC, Ozawa M, Shinya K, Zhong G, Hanson A, Katsura H, Watanabe S, Li C, Kawakami E, Yamada S, Kiso M, Suzuki Y, Maher EA, Neumann G, Kawaoka Y 2012 Experimental adaptation of an influenza H5 HA confers respiratory droplet transmission to a reassortant H5 HA/H1N1 virus in ferrets. Nature 486:420–428. 22722205 10.1038/nature10831 PMC3388103

[5] Zhang Y, Zhang Q, Kong H, Jiang Y, Gao Y, Deng G, Shi J, Tian G, Liu L, Liu J, Guan Y, Bu Z, Chen H 2013 H5N1 hybrid viruses bearing 2009/H1N1 virus genes transmit in guinea pigs by respiratory droplet. Science 340:1459–1463. doi:10.1126/science.1229455. 23641061

[6] Hatta M, Gao P, Halfmann P, Kawaoka Y 2001 Molecular basis for high virulence of Hong Kong H5N1 influenza A viruses. Science 293:1840–1842. doi:10.1126/science.1062882. 11546875

[7] Seo SH, Hoffmann E, Webster RG 2002 Lethal H5N1 influenza viruses escape host anti-viral cytokine responses. Nat Med 8:950–954. doi:10.1038/nm757. 12195436

[8] Li Z, Chen H, Jiao P, Deng G, Tian G, Li Y, Hoffmann E, Webster RG, Matsuoka Y, Yu K 2005 Molecular basis of replication of duck H5N1 influenza viruses in a mammalian mouse model. J Virol 79:12058–12064. doi:10.1128/JVI.79.18.12058-12064.2005. 16140781 PMC1212590

[9] Salomon R, Franks J, Govorkova EA, Ilyushina NA, Yen HL, Hulse-Post DJ, Humberd J, Trichet M, Rehg JE, Webby RJ, Webster RG, Hoffmann E 2006 The polymerase complex genes contribute to the high virulence of the human H5N1 influenza virus isolate A/Vietnam/1203/04. J Exp Med 203:689–697. doi:10.1084/jem.20051938. 16533883 PMC2118237

[10] Chen H, Bright RA, Subbarao K, Smith C, Cox NJ, Katz JM, Matsuoka Y 2007 Polygenic virulence factors involved in pathogenesis of 1997 Hong Kong H5N1 influenza viruses in mice. Virus Res 128:159–163. doi:10.1016/j.virusres.2007.04.017. 17521765

[11] Jiao P, Tian G, Li Y, Deng G, Jiang Y, Liu C, Liu W, Bu Z, Kawaoka Y, Chen H 2008 A single-amino-acid substitution in the NS1 protein changes the pathogenicity of H5N1 avian influenza viruses in mice. J Virol 82:1146–1154. doi:10.1128/JVI.01698-07. 18032512 PMC2224464

[12] Song MS, Pascua PN, Lee JH, Baek YH, Lee OJ, Kim CJ, Kim H, Webby RJ, Webster RG, Choi YK 2009 The polymerase acidic protein gene of influenza a virus contributes to pathogenicity in a mouse model. J Virol 83:12325–12335. doi:10.1128/JVI.01373-09. 19793828 PMC2786751

[13] Fan S, Deng G, Song J, Tian G, Suo Y, Jiang Y, Guan Y, Bu Z, Kawaoka Y, Chen H 2009 Two amino acid residues in the matrix protein M1 contribute to the virulence difference of H5N1 avian influenza viruses in mice. Virology 384:28–32. doi:10.1016/j.virol.2008.11.044. 19117585

[14] Song J, Feng H, Xu J, Zhao D, Shi J, Li Y, Deng G, Jiang Y, Li X, Zhu P, Guan Y, Bu Z, Kawaoka Y, Chen H 2011 The PA protein directly contributes to the virulence of H5N1 avian influenza viruses in domestic ducks. J Virol 85:2180–2188. doi:10.1128/JVI.01975-10. 21177821 PMC3067757

[15] Fan S, Hatta M, Kim JH, Le MQ, Neumann G, Kawaoka Y 2014 Amino acid changes in the influenza A virus PA protein that attenuate avian H5N1 viruses in mammals. J Virol 88:13737–13746. doi:10.1128/JVI.01081-14. 25231317 PMC4248959

[16] Fan S, Hatta M, Kim JH, Halfmann P, Imai M, Macken CA, Le MQ, Nguyen T, Neumann G, Kawaoka Y 2014 Novel residues in avian influenza virus PB2 protein affect virulence in mammalian hosts. Nat Commun 5:5021. doi:10.1038/ncomms6021. 25289523 PMC5841464

[17] Song J, Xu J, Shi J, Li Y, Chen H 2015 Synergistic effect of S224P and N383D substitutions in the PA of H5N1 avian influenza virus contributes to mammalian adaptation. Sci Rep 5:10510. doi:10.1038/srep10510. 26000865 PMC4441148

[18] Kawaoka Y, Webster RG 1988 Sequence requirements for cleavage activation of influenza virus hemagglutinin expressed in mammalian cells. Proc Natl Acad Sci U S A 85:324–328. doi:10.1073/pnas.85.2.324. 2829180 PMC279540

[19] Fodor E, Devenish L, Engelhardt OG, Palese P, Brownlee GG, Garcia-Sastre A 1999 Rescue of influenza A virus from recombinant DNA. J Virol 73:9679–9682. 10516084 10.1128/jvi.73.11.9679-9682.1999 PMC113010

[20] Neumann G, Watanabe T, Ito H, Watanabe S, Goto H, Gao P, Hughes M, Perez DR, Donis R, Hoffmann E, Hobom G, Kawaoka Y 1999 Generation of influenza A viruses entirely from cloned cDNAs. Proc Natl Acad Sci U S A 96:9345–9350. doi:10.1073/pnas.96.16.9345. 10430945 PMC17785

[21] Hoffmann E, Neumann G, Kawaoka Y, Hobom G, Webster RG 2000 A DNA transfection system for generation of influenza A virus from eight plasmids. Proc Natl Acad Sci U S A 97:6108–6113. doi:10.1073/pnas.100133697. 10801978 PMC18566

[22] Zhang Q, Shi J, Deng G, Guo J, Zeng X, He X, Kong H, Gu C, Li X, Liu J, Wang G, Chen Y, Liu L, Liang L, Li Y, Fan J, Wang J, Li W, Guan L, Li Q, Yang H, Chen P, Jiang L, Guan Y, Xin X, Jiang Y, Tian G, Wang X, Qiao C, Li C, Bu Z, Chen H 2013 H7N9 influenza viruses are transmissible in ferrets by respiratory droplet. Science 341:410–414. doi:10.1126/science.1240532. 23868922

[23] Gabriel G, Dauber B, Wolff T, Planz O, Klenk HD, Stech J 2005 The viral polymerase mediates adaptation of an avian influenza virus to a mammalian host. Proc Natl Acad Sci U S A 102:18590–18595. doi:10.1073/pnas.0507415102. 16339318 PMC1317936

[24] Chen LM, Davis CT, Zhou H, Cox NJ, Donis RO 2008 Genetic compatibility and virulence of reassortants derived from contemporary avian H5N1 and human H3N2 influenza A viruses. PLoS Pathog 4:e1000072. doi:10.1371/journal.ppat.1000072. 18497857 PMC2374906

[25] Li C, Hatta M, Nidom CA, Muramoto Y, Watanabe S, Neumann G, Kawaoka Y 2010 Reassortment between avian H5N1 and human H3N2 influenza viruses creates hybrid viruses with substantial virulence. Proc Natl Acad Sci U S A 107:4687–4692. doi:10.1073/pnas.0912807107. 20176961 PMC2842136

[26] Binh NT, Wakai C, Kawaguchi A, Nagata K 2014 Involvement of the N-terminal portion of influenza virus RNA polymerase subunit PB1 in nucleotide recognition. Biochem Biophys Res Commun 443:975–979. doi:10.1016/j.bbrc.2013.12.071. 24361882

[27] González S, Ortin J 1999 Characterization of influenza virus PB1 protein binding to viral RNA: two separate regions of the protein contribute to the interaction domain. J Virol 73:631–637. 9847368 10.1128/jvi.73.1.631-637.1999 PMC103869

[28] Kerry PS, Willsher N, Fodor E 2008 A cluster of conserved basic amino acids near the C-terminus of the PB1 subunit of the influenza virus RNA polymerase is involved in the regulation of viral transcription. Virology 373:202–210. doi:10.1016/j.virol.2007.11.030. 18191435

[29] Biswas SK, Nayak DP 1994 Mutational analysis of the conserved motifs of influenza A virus polymerase basic protein 1. J Virol 68:1819–1826. 8107244 10.1128/jvi.68.3.1819-1826.1994 PMC236644

[30] Chu C, Fan S, Li C, Macken C, Kim JH, Hatta M, Neumann G, Kawaoka Y 2012 Functional analysis of conserved motifs in influenza virus PB1 protein. PLoS One 7:e36113. doi:10.1371/journal.pone.0036113. 22615752 PMC3352917

[31] González S, Zurcher T, Ortin J 1996 Identification of two separate domains in the influenza virus PB1 protein involved in the interaction with the PB2 and PA subunits: a model for the viral RNA polymerase structure. Nucleic Acids Res 24:4456–4463. doi:10.1093/nar/24.22.4456. 8948635 PMC146260

[32] Perez DR, Donis RO 2001 Functional analysis of PA binding by influenza A virus PB1: effects on polymerase activity and viral infectivity. J Virol 75:8127–8136. doi:10.1128/JVI.75.17.8127-8136.2001. 11483758 PMC115057

[33] Poole EL, Medcalf L, Elton D, Digard P 2007 Evidence that the C-terminal PB2-binding region of the influenza A virus PB1 protein is a discrete alpha-helical domain. FEBS Lett 581:5300–5306. doi:10.1016/j.febslet.2007.10.025. 17967456

[34] Naffakh N, Tomoiu A, Rameix-Welti MA, van der Werf S 2008 Host restriction of avian influenza viruses at the level of the ribonucleoproteins. Annu Rev Microbiol 62:403–424. doi:10.1146/annurev.micro.62.081307.162746. 18785841

[35] Sugiyama K, Obayashi E, Kawaguchi A, Suzuki Y, Tame JRH, Nagata K, Park SY 2009 Structural insight into the essential PB1-PB2 subunit contact of the influenza virus RNA polymerase. EMBO J 28:1803–1811. doi:10.1038/emboj.2009.138. 19461581 PMC2699363

[36] Li J, Li Y, Hu Y, Chang G, Sun W, Yang Y, Kang X, Wu X, Zhu Q 2011 PB1-mediated virulence attenuation of H5N1 influenza virus in mice is associated with PB2. J Gen Virol 92:1435–1444. doi:10.1099/vir.0.030718-0. 21367983

[37] Binh NT, Wakai C, Kawaguchi A, Nagata K 2013 The N-terminal region of influenza virus polymerase PB1 adjacent to the PA binding site is involved in replication but not transcription of the viral genome. Front Microbiol 4:398. doi:10.3389/fmicb.2013.00398. 24391632 PMC3866587

[38] He X, Zhou J, Bartlam M, Zhang R, Ma J, Lou Z, Li X, Li J, Joachimiak A, Zeng Z, Ge R, Rao Z, Liu Y 2008 Crystal structure of the polymerase PA(C)-PB1(N) complex from an avian influenza H5N1 virus. Nature 454:1123–1126. doi:10.1038/nature07120. 18615018

[39] Zhang Y, Zhang Q, Gao Y, He X, Kong H, Jiang Y, Guan Y, Xia X, Shu Y, Kawaoka Y, Bu Z, Chen H 2012 Key molecular factors in hemagglutinin and PB2 contribute to efficient transmission of the 2009 H1N1 pandemic influenza virus. J Virol 86:9666–9674. doi:10.1128/JVI.00958-12. 22740390 PMC3446561

[40] Zhu Q, Yang H, Chen W, Cao W, Zhong G, Jiao P, Deng G, Yu K, Yang C, Bu Z, Kawaoka Y, Chen H 2008 A naturally occurring deletion in its NS gene contributes to the attenuation of an H5N1 swine influenza virus in chickens. J Virol 82:220–228. doi:10.1128/JVI.00978-07. 17942562 PMC2224367

[41] Tarendeau F, Boudet J, Guilligay D, Mas PJ, Bougault CM, Boulo S, Baudin F, Ruigrok RW, Daigle N, Ellenberg J, Cusack S, Simorre JP, Hart DJ 2007 Structure and nuclear import function of the C-terminal domain of influenza virus polymerase PB2 subunit. Nat Struct Mol Biol 14:229–233. doi:10.1038/nsmb1212. 17310249

